# The Environmental Plasticity of Diverse Body Color Caused by Extremely Long Photoperiods and High Temperature in *Saccharosydne procerus* (Homoptera: Delphacidae)

**DOI:** 10.3389/fphys.2016.00401

**Published:** 2016-09-12

**Authors:** Haichen Yin, Qihao Shi, Muhammad Shakeel, Jing Kuang, Jianhong Li

**Affiliations:** ^1^Department of Plant Protection, College of Plant Science and Technology, Huazhong Agricultural UniversityWuhan, China; ^2^Wuhan Vegetable Research InstituteWuhan, China

**Keywords:** green slender planthopper, life traits, melanization, plasticity, thermal melanism hypothesis

## Abstract

Melanization reflects not only body color variation but also environmental plasticity. It is a strategy that helps insects adapt to environmental change. Different color morphs may have distinct life history traits, e.g., development time, growth rate, and body weight. The green slender planthopper *Saccharosydne procerus* (Matsumura) is the main pest of water bamboo (*Zizania latifolia*). This insect has two color morphs. The present study explored the influence of photoperiod and its interaction with temperature in nymph stage on adult melanism. Additionally, the longevity, fecundity, mating rate, and hatching rate of *S. procerus* were examined to determine whether the fitness of the insect was influenced by melanism under different temperature and photoperiod. The results showed that a greater number of melanic morphs occurred if the photoperiod was extremely long. A two-factor ANOVA showed that temperature and photoperiod both have a significant influence on melanism. The percentages of variation explained by these factors were 45.53 and 48.71%, respectively. Moreover, melanic morphs had greater advantages than non-melanic morphs under an environmental regime of high temperatures and a long photoperiod, whereas non-melanic morphs were better adapted to cold temperatures and a short photoperiod. These results cannot be explained by the thermal melanism hypothesis. Thus, it may be unavailable to seek to explain melanism in terms of only one hypothesis.

## Introduction

**Figure 1:**
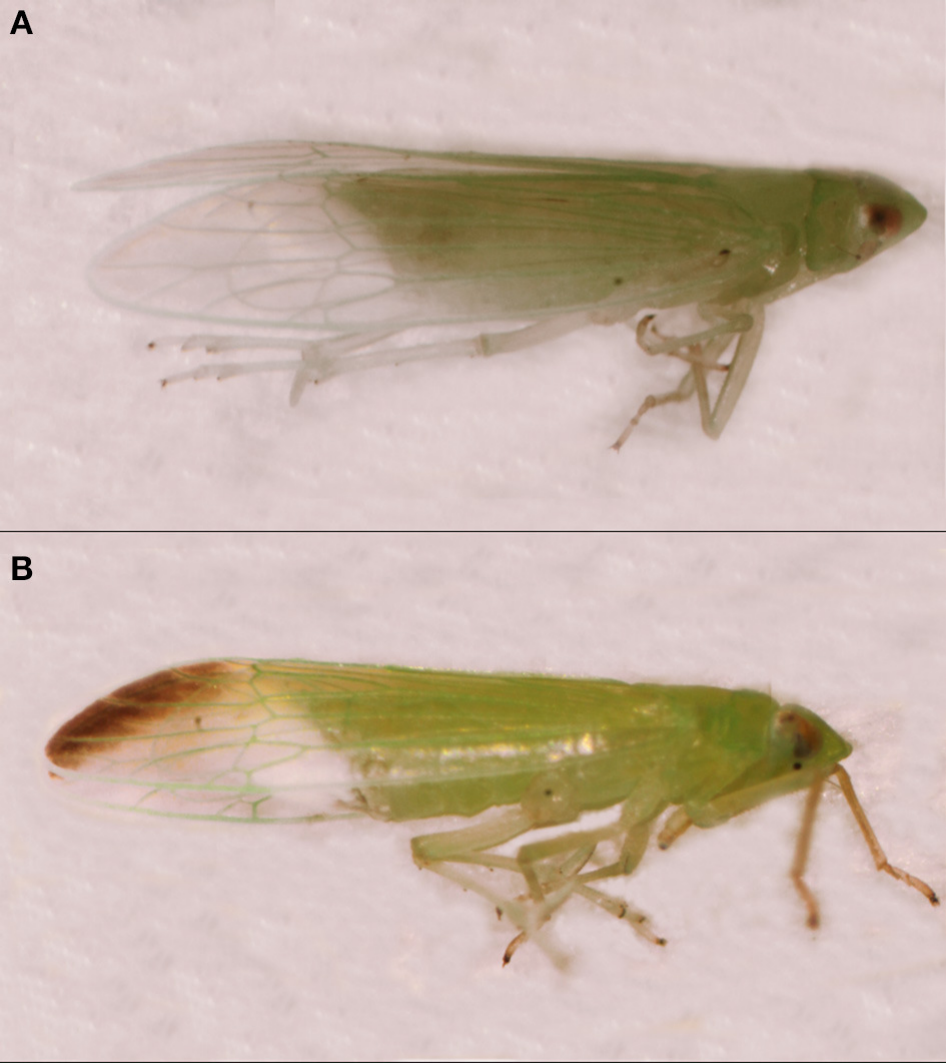
**The non-melanic and melanic *Saccharosydne procerus*. (A)** Non-melanic morph and **(B)** Melanic morph. Figure adapted from Yin et al. (2015).

The green slender planthopper *Saccharosydne procerus* (Matsumura) is the principal pest of water bamboo (*Zizania latifolia*) and has also been shown to damage rice (*Oryza sativa*) in some East Asian countries such as China and Vietnam. Some green slender planthoppers have a black spot on the terminus of the forewing (Figure 1). These phenotypes represent the melanic morph of the species (Yin et al., 2015). The black spot appears after emergence. Melanism in this species does not change over the adult stage. Previous studies have shown that the proportion of adult melanic morphs is influenced by the environmental temperature in the nymph stage. Under high- temperature conditions, there are more melanic morphs (Mao, 2008; Kuang, 2012). This pattern contradicts the thermal melanism hypothesis (TMH). This hypothesis predicts that dark individuals with low skin reflectance will heat faster than lighter individuals so that melanic morphs will have an advantage in cool climates and under a short photoperiod (Clusella-Trullas et al., 2008).

To date, the TMH has been confirmed by a large number of studies. For example, cuticle melanism of the ground cricket *Allonemobius socius* had a positive association with season length (Fedorka et al., 2013); body melanization of *Drosophila melanogaster* showed significant correlations with temperature, and individuals fed in cool conditions were darker (Parkash et al., 2010); additionally, the frequency of the melanic two-spotted ladybird *Adalia bipunctata* decreased as a result of an increase in local ambient spring temperatures (de Jong and Brakefield, 1998).

Melanization not only reflects body color variation, but also environmental plasticity (Karlsson and Forsman, 2010); it is a strategy used by insects to adapt to the environmental alterations (van't Hof et al., 2011). According to the TMH, dark individuals absorb heat faster than lighter ones (Clusella-Trullas et al., 2008). Hence, different body colors lead to variation in body temperatures, and such variation may affect many life history traits (Su et al., 2013), e.g., development time, growth rate, and body weight (Cotter et al., 2008; Ma et al., 2008).

Although the TMH is widely accepted, we can still identify some counterexamples. Female *Tetranychus* spider mites did not show dark cuticular pigmentation when exposed to short-day and low-temperature conditions (Ito et al., 2013). In some cases, melanic morphs were observed in some tropical areas and warmer habitats (Rajpurohit et al., 2008). These studies indicate a complex effect of the environmental temperature on insect melanism. In view of the complicated mechanism involved, the TMH must not be the only explanation of melanism. Therefore, it cannot provide a universal explanation for melanism (Wittkopp et al., 2003).

Although much information about melanism in insects is available to date, little is known about melanism in aquatic vegetable pests such as *S. procerus*. Melanism in this specie has been shown to be influenced by temperature. Its pattern is opposite to the one predicted by the TMH (Mao, 2008; Kuang, 2012). Hence, it is interesting to study the variation of the environmental plasticity caused by melanism. Additionally, we suggest that it would be worthwhile to study the influence of photoperiod on melanism and the relationship between the effects of temperature and photoperiod on melanism.

The effect of photoperiod on melanism was investigated in this study. A two-factor ANOVA was employed to analyze the relationship between the influences of temperature and photoperiod on melanism. Moreover, the longevity, fecundity, mating rate, and hatching rate of *S. procerus* were examined to investigate whether the fitness of the insect in the studied environments was influenced by melanism under variations in temperature and photoperiod. In particular, as melanism persists in the adult stage, we predicted that some life history traits of the two morphs would show contrasting differences in the adult stage. The variation in the proportion of melanism may be caused by differences in the adaptations of the two morphs, or melanism may be a way for *S. procerus* to adjust in the environmental changes. Because *S. procerus* faces pressure caused by simultaneous variations in temperature and photoperiod in the natural environment, we consider it of interest to explore the relative significance of these two factors for melanism.

## Materials and methods

### Rearing of *S. procerus*

In this study, *S. procerus* were reared on water bamboo grown in the greenhouse of Huazhong Agriculture University, Wuhan (N30°28′, E114°21′), China. To provide living conditions similar to the natural habitat, the greenhouse was only covered by a gauze net. The *S. procerus* in the present study were not exposed to any pesticides. Hence, they did not face any selection pressure from pesticides.

### The effect of photoperiod on melanism

Leaves of water bamboo with spawning marks were collected in the greenhouse. These eggs were placed in an artificial climate box at 26°C with L:D 16:8 h photoperiod. Four photoperiod treatments (8:16, 12:12, 16:8, 20:4; 26°C) were employed to explore the effect of photoperiod on melanism. Five nymphs were placed on one water bamboo leaf in a glass tube (14 × 1.5 cm). Each replicate contained 25 nymphs and each treatment contained four replicates. Therefore, 100 individuals were used in one treatment.

### The contribution of photoperiod and temperature to variations in melanism

To compare the relative influence of photoperiod and temperature on melanism, four treatments (20:4, 30°C; 20:4, 22°C; 8:16, 30°C; 8:16, 22°C) were employed. The egg collection and rearing methods used were stated above.

### Observation of life traits

Water bamboo leaves with spawning marks were gathered from the field to facilitate the collection of eggs of green slender planthoppers. These eggs were placed in an artificial climate box at 26°C and L:D = 16:8 photoperiod to feed for one generation.

A pair of melanic adults or non-melanic adults was placed in one glass tube (14 × 1.5 cm) with one water bamboo leaf. Each experimental tube received a selected combination of temperature and photoperiod; three temperatures (22°C, 26°C, and 30°C, all at L:D = 16:8 photoperiod) and three photoperiods (12:12, 16:8, and 20:4; all at 26°C) were used in this experiment. The experiment was performed to compare selected life history traits of adults, namely, adult longevity, fecundity, mating rate, hatching rate, pre-oviposition period, and egg stage duration. All the data were recorded daily. The leaves were changed once a day, and 100 melanic adults and 100 non-melanic adults were employed for each treatment. The rationale for selecting these temperatures was that under these treatments, the melanism proportion was found to differ significantly; moreover, a previous study showed that these conditions were suitable for *S. procerus* (Kuang, 2012).

### Statistical analysis

In the present study, one-way ANOVA and pairwise assessment using the Multiple Range Test were applied to analyze the effect of photoperiod on melanism and the influence of the studied factors on life history traits. A two-factor ANOVA was employed to analyze the contributions of photoperiod and temperature to variation in melanism. All analyses were conducted with SPSS Statistics 17.0.

## Results

### The effect of photoperiod on melanism

**Figure 2:**
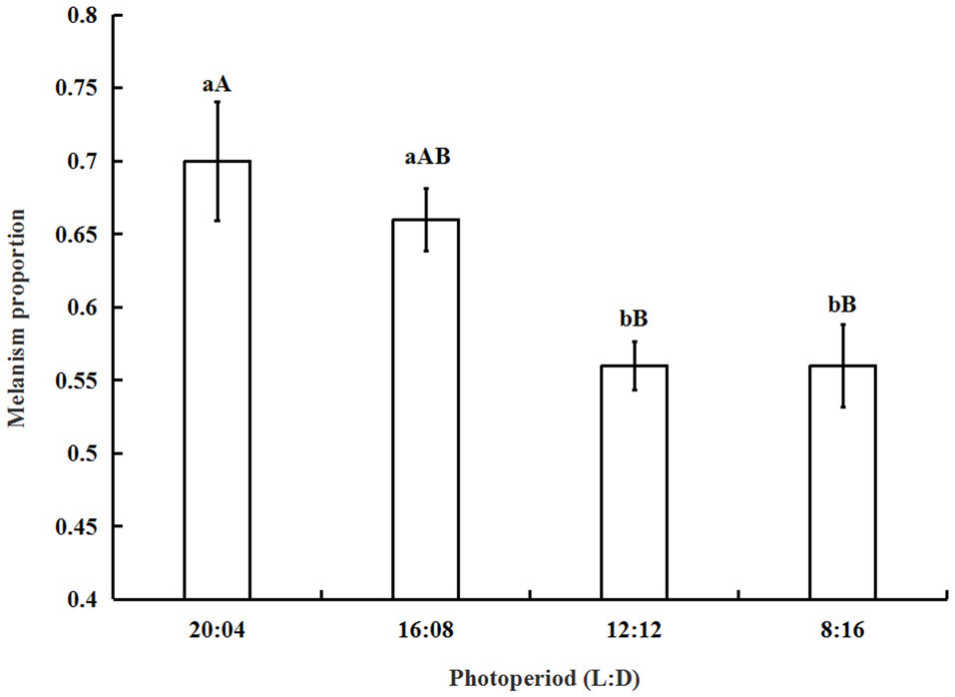
**The proportion of melanism (mean ± SD) under different photoperiods**. The proportion of melanism increased when the light period become longer. The proportion of melanism under 16:08 was significantly higher than that under 12:12 (*P* = 0.0341) or 8:16 (*P* = 0.0279). The same letter represent there is no significant differences in statistics. The capital letters represent the level of 1%, the lower-case letters represent the level of 5%.

The proportion of melanic morphs increased from 0.56 ± 0.03 under short photoperiod (L:D = 8:16) to 0.7 ± 0.04 under long photoperiod (L:D = 20:04) (Figure 2). The results showed that long photoperiod is beneficial to improve the number of melanic morphs. The analysis of one-way ANOVA showed that the proportion of melanism under 16:08 was significantly higher than that under 12:12 (*P* = 0.0341) and 8:16 (*P* = 0.0279). The differences between the melanism proportions under 20:04 and 16:08 were not significant (*P* > 0.05). The proportion of melanism under 12:12 did not differ significantly from that under 8:16 (*P* > 0.05).

### The contributions of photoperiod and temperature to variations in melanism

**Figure 3:**
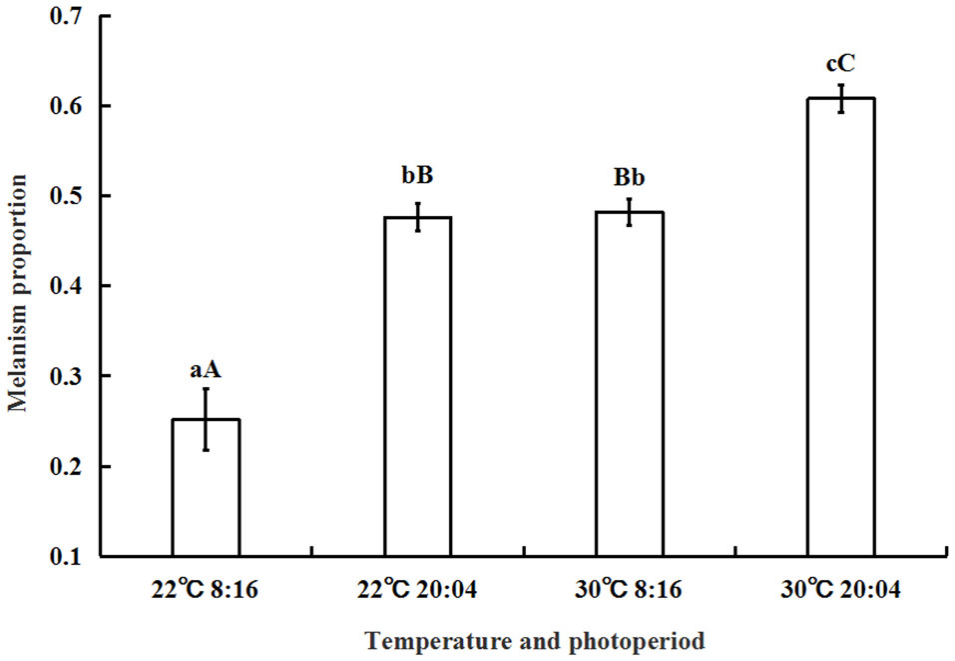
**The effect of temperature, photoperiod, and their interaction on the proportion of melanism (mean ± SD)**. A two-factor ANOVA showed that temperature and photoperiod both have a significant influence on melanism (*P* < 0.01). The proportions of total variance explained by photoperiod and temperature were 45.53 and 48.71%, respectively. The same letter represent there is no significant differences in statistics. The capital letters represent the level of 1%, the lower-case letters represent the level of 5%.

Under high temperature and extremely long photoperiod (30°C, L:D = 20:04) the melanism proportion was 0.61 ± 0.01 while this proportion decreased to 0.25 ± 0.03 under 22°C at L:D = 8:16. A two-factor ANOVA showed that temperature and photoperiod both have significant influences on melanism (*P* < 0.01). The percentages of variation in melanism explained by photoperiod and temperature were 45.53% and 48.71%, respectively. The interaction between photoperiod and temperature explained 3.57% of the total variation in melanism (Figure 3).

### Adult longevity

Under 22°C, the male adult longevity of two color morphs was 11.3 ± 2.54 (melanic morphs) and 18.8 ± 1.79 (non-melanic morphs), respectively, while, when the temperature increased to 30°C, it was 13.8 ± 0.84 (melanic morphs) and 10.2 ± 3.03 (non-melanic morphs), respectively. The male adult longevity of two morphs varied from 5.4 ± 1.34 (melanic morphs) and 13.8 ± 1.92 (non-melanic morphs) under 12:12 to 14.7 ± 1.51 (melanic morphs) and 10.0 ± 1.84 (non-melanic morphs) under 20:04.

Similarly under 22°C the female adult longevity of two color morphs was respectively 10.0 ± 1.06 (melanic morphs) and 16.2 ± 2.17 (non-melanic morphs), while when temperature increased to 30°C, it was 12.0 ± 1.87 (melanic morphs) and 8.8 ± 0.45 (non-melanic morphs), respectively. With the change of photoperiod, the female adult longevity of two morphs also changed from 5.25 ± 0.90 (melanic morphs) and 15.4 ± 1.52 (non-melanic morphs) under 12:12 to 14.1 ± 0.89 (melanic morphs) and 8.45 ± 1.23 (non-melanic morphs) under 20:04.

One-way ANOVA showed that under all temperature and photoperiod treatments, there were no significant differences between the longevity of male adults and that of female adults (*P* > 0.05). The longevity of non-melanic male and female adults was significantly longer than that of melanic male adults (*P* = 0.0006) and female adults (*P* = 0.0004) at 22°C. At 26°C, the longevity of non-melanic adults did not differ significantly from that of melanic morphs (*P* > 0.05). The longevity of melanic male and female adults was significantly longer than that of non-melanic male adults (*P* = 0.0337) and female adults (*P* = 0.0059) at 30°C.

**Figure 4:**
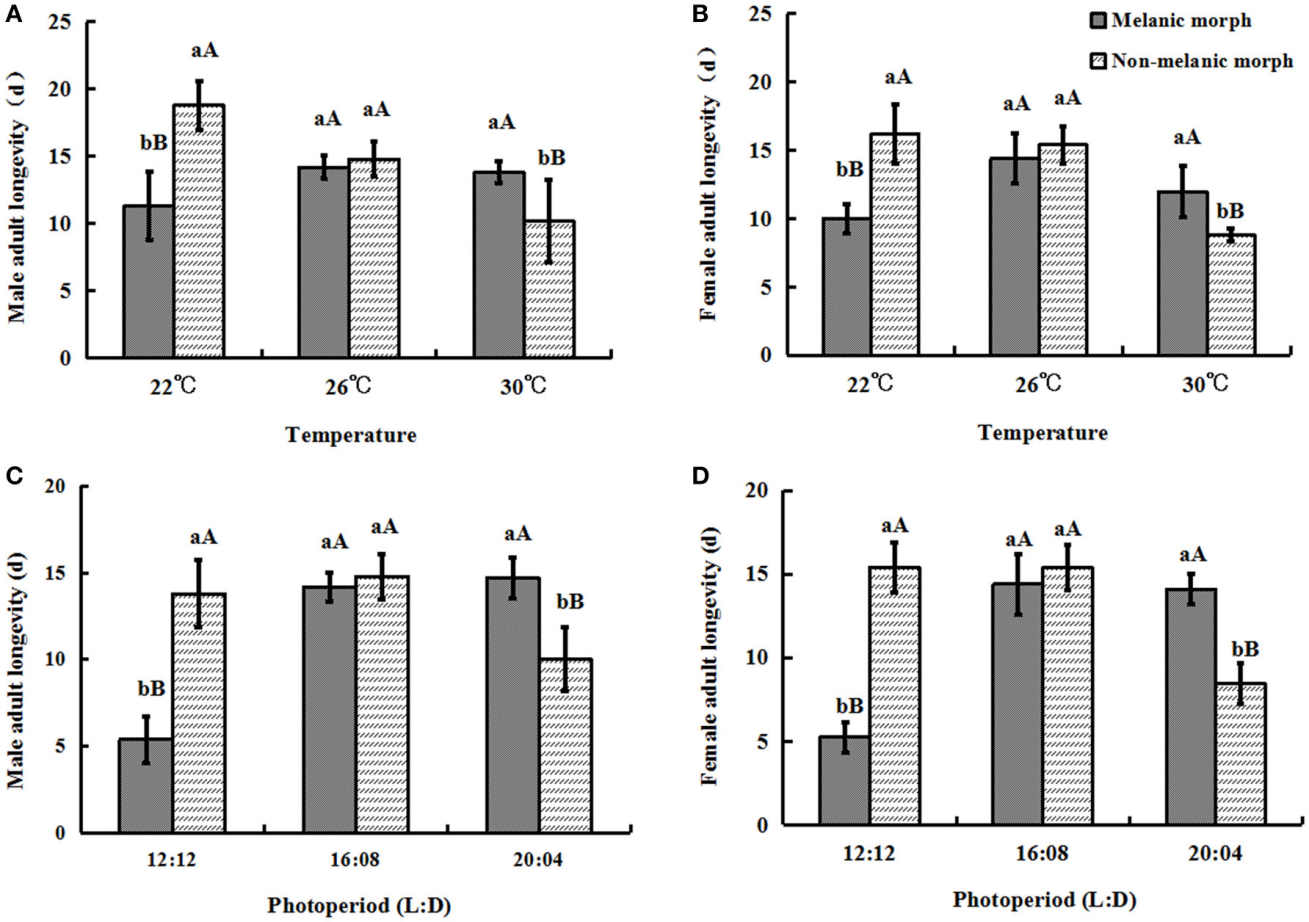
**Adult longevity (mean ± SD) of *S. procerus*. (A)** Male adult longevity at different temperatures. **(B)** Female adult longevity at different temperatures. **(C)** Male adult longevity under different photoperiods. **(D)** Female adult longevity under different photoperiods. Under all temperature and photoperiod treatments, there were no significant differences between the longevity of male adults and that of female adults (*P* > 0.05). The longevity of melanic adults was significantly longer than that of non-melanic adults at 30°C and 20:04 but shorter than that of non-melanic adults at 22°C and 12:12 (*P* < 0.01). The same letter represent there is no significant differences in statistics. The capital letters represent the level of 1%, the lower-case letters represent the level of 5%.

Under the 12:12 photoperiod, non-melanic males and females had a greater longevity than that of melanic males (*P* < 0.0001) and females (*P* < 0.0001). Melanic males and females had a longer longevity than non-melanic males (*P* = 0.0013) and females (*P* < 0.0001) under the 20:04 photoperiod. There were no significant differences among the treatment groups under the 16:08 photoperiod (*P* > 0.05) (Figure 4).

### Fecundity per female

The fecundity per female of melanic morphs varied from 27.0 ± 4.00 under 22°C and 48.4 ± 5.58 under 12:12 to 99.0 ± 21.84 under 30°C and 46.7 ± 1.10 under 20:04. For non-melanic morphs, fecundity per female changed from 72.0 ± 16.386 (22°C), 80.9 ± 16.98 (12:12) to 68.2 ± 5.67 (30°C), 35.5 ± 7.07 (20:04), respectively.

**Figure 5:**
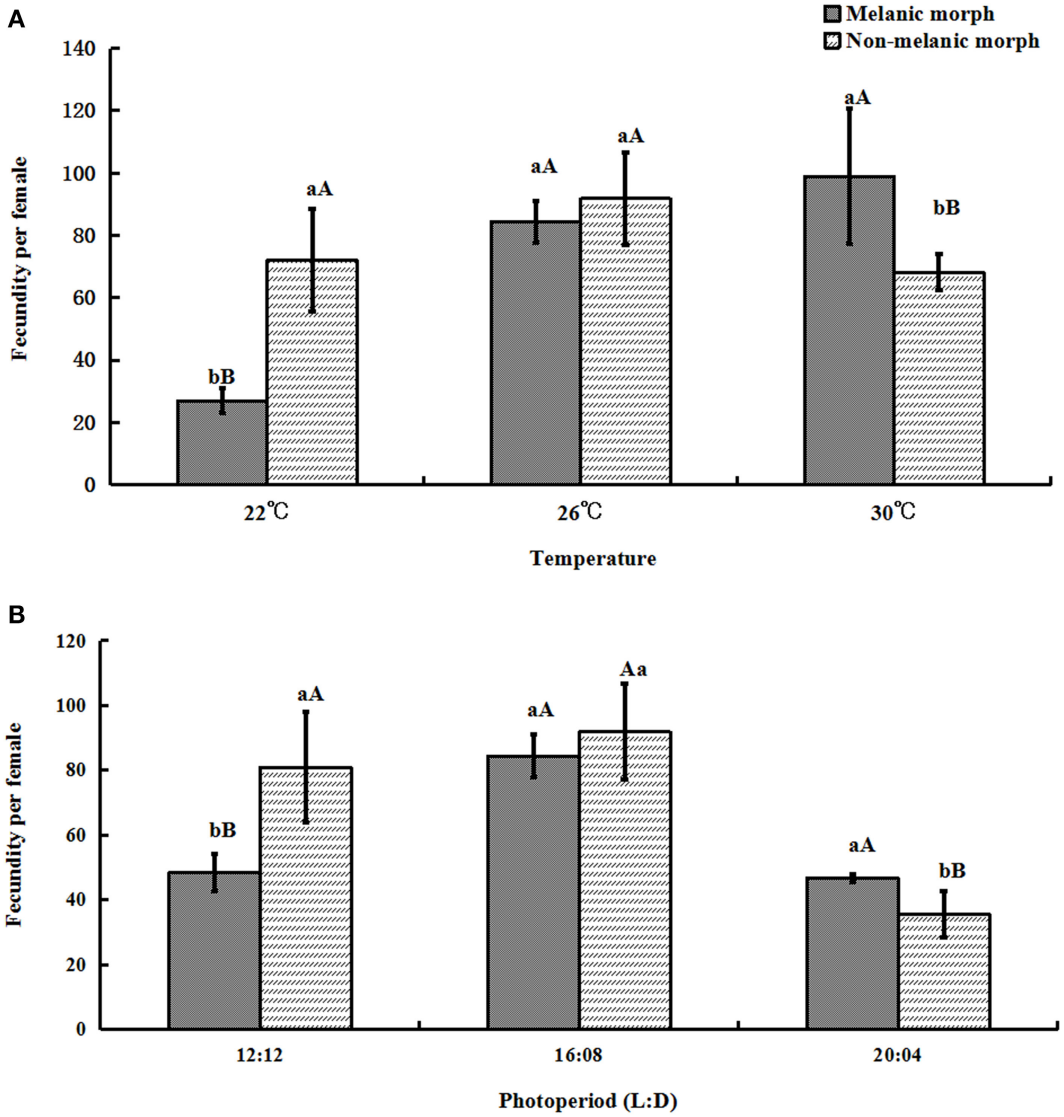
**The fecundity per female (mean ± SD) of *S. procerus*. (A)** The fecundity per female at different temperatures. **(B)** The fecundity per female under different photoperiods. The fecundity of melanic females was significantly higher than that of non-melanic females at 30°C and 20:04. It was lower than that of non-melanic females at 22°C and 12:12 (*P* < 0.01). The same letter represent there is no significant differences in statistics. The capital letters represent the level of 1%, the lower-case letters represent the level of 5%.

One-way ANOVA showed that the fecundity per female of melanic morphs was markedly higher than that of non-melanic morphs at 30°C (*P* = 0.0158) and 20:04 (*P* = 0.0081), and lower than that of non-melanic morphs at 22°C (*P* = 0.0003) and 12:12 (P = 0.0036). There were no statistically significant differences between the two morphs at 26°C or under 16:08 (*P* > 0.05) (Figure 5).

### Mating rate

If a female adult produced no offspring, that female was considered as failed to mate. When the temperature increased, the mating rate of melanic morphs varied from (66.0 ± 5.48)% to (60.0 ± 7.07)%, similarly, it was (92.0 ± 8.37)% under 22°C and decreased to (44.0 ± 5.48)% under 30°C for non-melanic morphs. Under 12:12, it was (52.502 ± 2.28)% (melanic morphs) and (84.446 ± 6.09)% (non-melanic morphs), when the daytime became longer it was (95.0 ± 6.09)% (melanic morphs) and (70.0 ± 6.85)% (non-melanic morphs), respectively, under 20:04.

**Figure 6:**
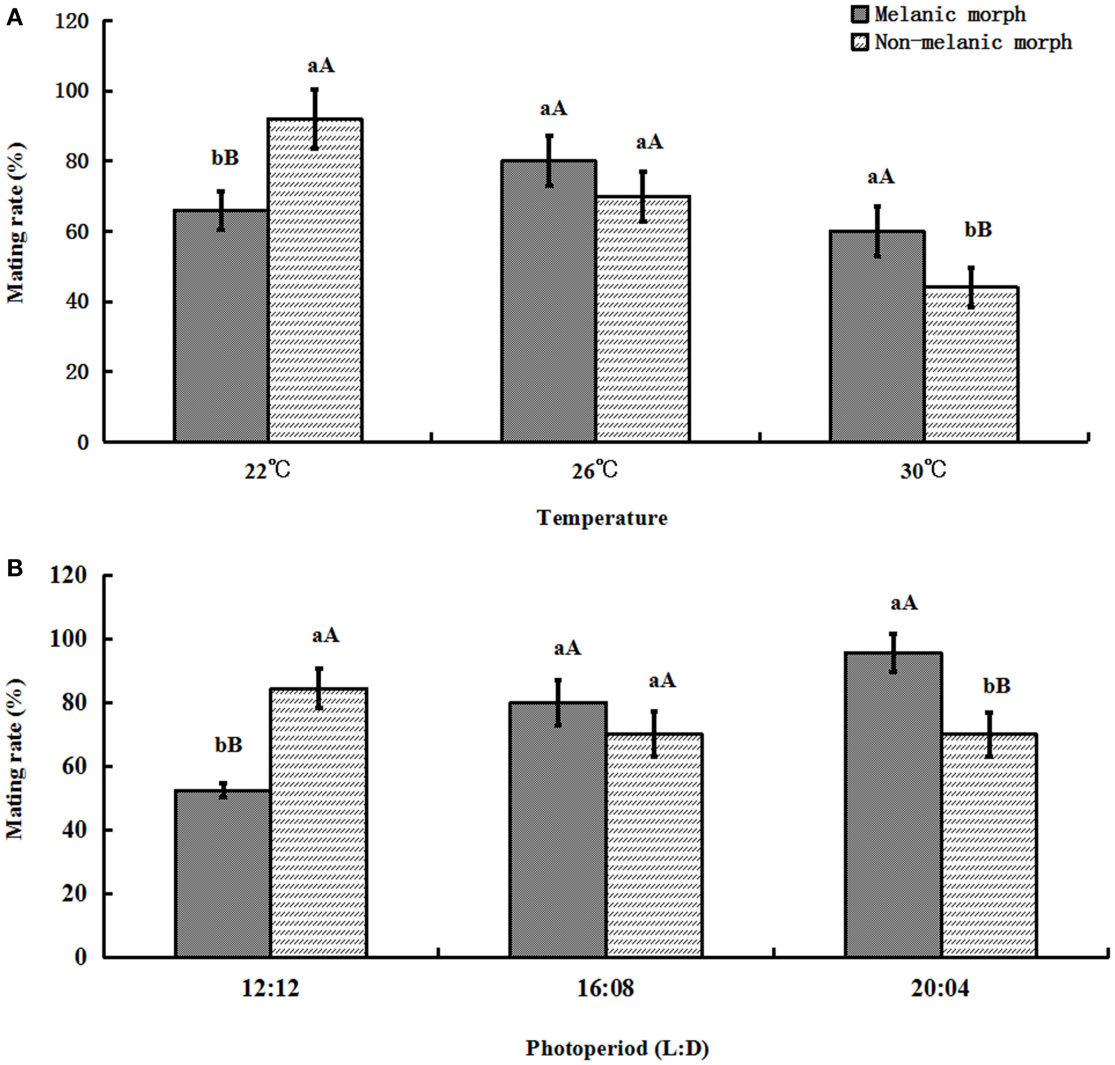
**Mating rate (%) (mean ± SD) of *S. procerus*. (A)** Mating rate (%) at different temperatures. **(B)** Mating rate (%) under different photoperiods. The mating rate of melanic morphs was significantly higher than that of non-melanic adults under 30°C and 20:04. It was lower than that of non-melanic adults at 22°C and 12:12 (*P* < 0.01). The same letter represent there is no significant differences in statistics. The capital letters represent the level of 1%, the lower-case letters represent the level of 5%.

One-way ANOVA showed that the mating rate of melanic morphs was significantly higher than that of non-melanic morphs at 30°C (*P* = 0.0039) and under 20:04 (*P* = 0.0002) and lower than that of non-melanic morphs at 22°C (*P* = 0.0004) and under 12:12 (*P* < 0.0001). There were no statistically significant differences between the two morphs at 26°C or under 16:08. (*P* > 0.05) (Figure 6).

### Hatching rate

When the temperature and daytime increased, the hatching rate of eggs produced by melanic morphs increased from (54.526 ± 10.18)% and (46.778 ± 7.44)% to (79.464 ± 7.61)% and (78.037 ± 4.42)%, while it decreased from (85.45 ± 4.54)% and (71.689 ± 4.97)% to (56.618 ± 8.95)% and (46.416 ± 5.79)% for non-melanic morphs, respectively.

**Figure 7:**
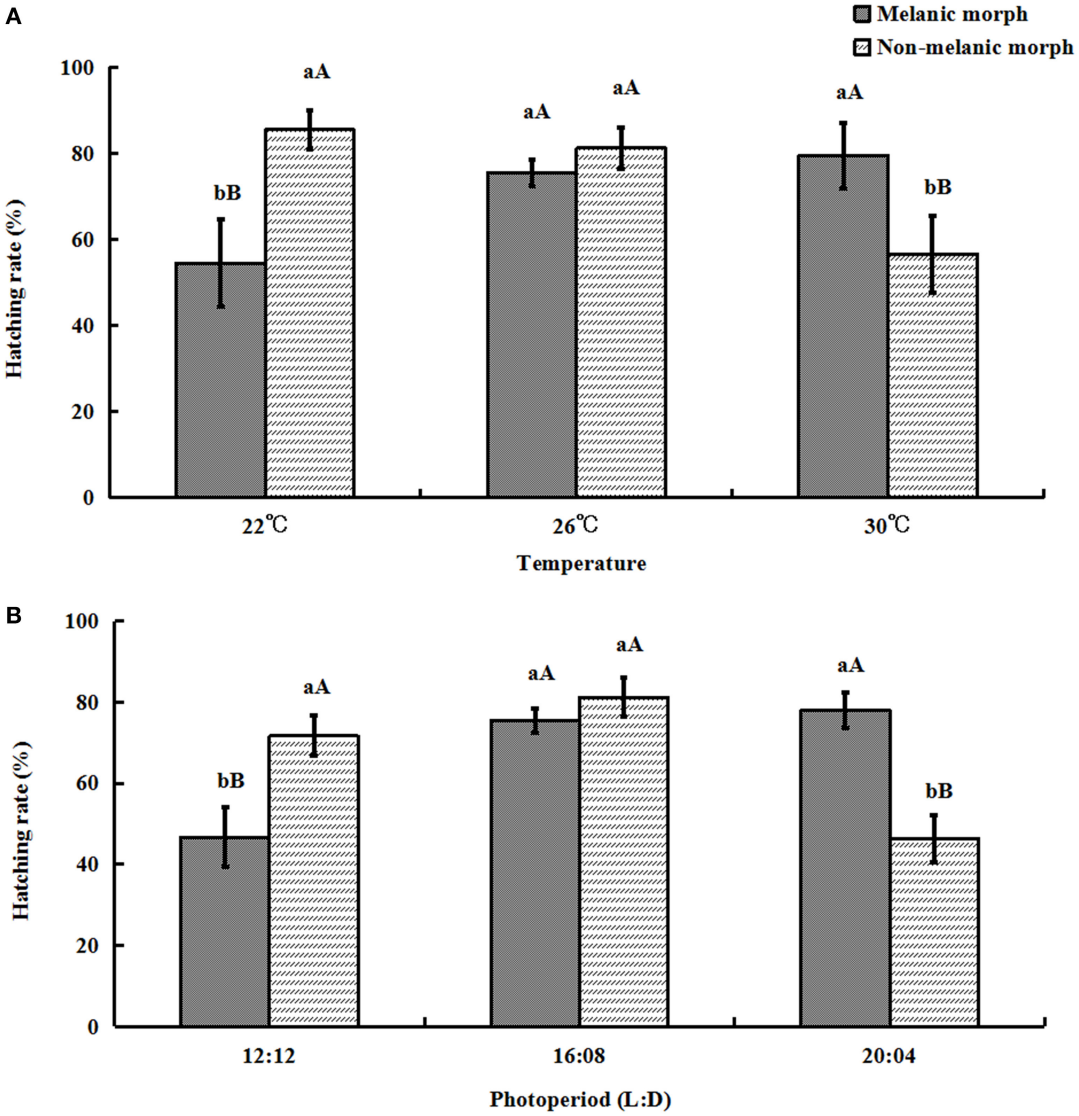
**Hatching rate (%) (mean ± SD) of *S. procerus*. (A)** Hatching rate at different temperatures. **(B)** Hatching rate under different photoperiods. The hatching rate of melanic morphs was significantly higher than that of non-melanic adults at 30°C and 20:04. It was lower than that of non-melanic adults at 22°C and 12:12 (*P* < 0.01). The same letter represent there is no significant differences in statistics. The capital letters represent the level of 1%, the lower-case letters represent the level of 5%.

One-way ANOVA showed that eggs produced by non-melanic adults had a significantly higher hatching rate than that of eggs produced by melanic adults at 22°C (*P* = 0.0003) and under 12:12 (*P* = 0.0003). The hatching rate of eggs produced by melanic adults was significantly higher than that of eggs produced by non-melanic adults at 30°C (*P* = 0.0025) and under 20:04 (*P* < 0.0001). No significant differences between two morphs were found at 26°C or under 16:08 (*P* > 0.05) (Figure 7).

### Preoviposition period and egg period

When the temperature increased from 22°C to 30°C, the pre-oviposition of melanic and non-melanic morphs varied from (6.6 ± 1.5477) d and (5.0 ± 1.7070) d to (4.0 ± 0.7071) d and (3.8 ± 0.4472). Egg period of melanic and non-melanic morphs varied from (15.0 ± 0.7701) d and (16.4 ± 1.3416) d to (10.2 ± 2.2804) d and (11.8 ± 1.7889) d. Pre-oviposition and egg period of two morphs both shortened when the temperature increased.

When the photoperiod increased from 12:12 to 20:04, the pre-oviposition of melanic and non-melanic morphs varied from (5.2 ± 0.7583) d and (5.0 ± 1.0000) d to (5.0 ± 1.5811) d and (5.1 ± 0.4472) d. Egg period of melanic and non-melanic morphs varied from (11.8 ± 1.3038) d and (10.7 ± 0.6708) d to (12.0 ± 0.7071) d and (11.1 ± 0.7416) d. Pre-oviposition and egg period of two morphs did not varied significantly under different photoperiod. One-way ANOVA showed that the preoviposition period and egg period had no significant differences between the two morphs (*P* > 0.05).

## Discussion

A previous study has shown that the proportion of melanism in *S. procerus* increased under higher temperature in the laboratory. This finding was also obtained in a field study, previous field investigation in four primary water bamboo producing areas showed that the proportion of melanic morphs was highest in the areas that had highest mean temperature, field investigations in Wuhan (N30°28′ and E114°21′), China from June to October 2012 also proved that high temperature improve the melanic proportion (Yin et al., 2015). In the present study, our data showed that photoperiod also had marked effects on melanism in *S. procerus* besides temperature. Notably, the photoperiod that was found to have a statistically significant effect on the proportion of melanism was an extreme case. Accordingly, we speculated that the influence of photoperiod might be not as strong as that of temperature. However, this speculation was not supported by a two-factor ANOVA. This analysis found that the percentages of variation in melanism explained by photoperiod and temperature were 45.53 and 48.71% respectively. Nevertheless, given that this photoperiod used in our laboratory study does not actually occur in the field, so the variation in the proportion of melanism in *S. procerus* in the principal areas of water bamboo (*Z. latifolia*) production in China should still be explained by the effect of temperature.

Several previous studies showed that the host-pathogen interaction can be influenced by the thermal environment (Cotter et al., 2008; Catalán et al., 2012). When a pathogen binds to a pattern recognition receptor, the prophenoloxidase-activating proteinase (PAP) will convert pro-phenoloxidase (PPO) to phenoloxidase (PO) (Yu et al., 2003). After this conversion, PO is involved in the synthesis of melanin with the oxidation of L-Dopa or dopamine, and this process contributes to both pathogen defense and melanism (Fedorka et al., 2013). When faced with a greater risk of infection, individuals will invest more in the immune system (Wilson and Reeson, 1998). Due to the close connection between melanization and immune response, this investment will influence body color (Mills, 2012). Similarly, we speculated that the body color of *S. procerus* may change in the same way. According to previous study, increased temperature is usually associated with the increase in infectious diseases (Roulin, 2014). Hence, melanic morphs of *S. procerus* appeared, which may be due to the higher PO activity and melanin synthesis caused by greater infectious risk under high temperature (Wilson et al., 2001; Eleftherianos and Revenis, 2011). Additionally, the UV-absorbing properties of melanin are believed to be involved in protecting the organism from UV-induced DNA damage (Li et al., 2015). Indeed, a previous study showed that UV-exposed animals usually had a higher melanin content and became darker (Debecker et al., 2015). Therefore, we speculated that the melanism of *S. procerus* induced by extremely long photoperiods serves to facilitate UV protection. Moreover, we infer that the multiple function of melanin may not only change the body color of *S. procerus*, but also the environmental fitness.

In this study, differences between the life history traits of the two studied morphs under different conditions were compared to explore the influence of melanism on the fitness of *S. procerus*. We found that melanic morphs enjoyed a greater advantage than non-melanic morphs in a hot environment and under extremely long photoperiod, whereas, non-melanic morphs were better adapted to cold conditions and relatively short photoperiods. These results showed that the melanism of *S. procerus* is expressed not only as variation in body color but also in fitness differences in the studied environments. Given that melanism can be induced in hot environments and under extremely long photoperiod, we think that melanism may be a strategy by which *S. procerus* adapts to environmental alterations. However, the thermal plasticity of *S. procerus* cannot be explained by the TMH. Insects face complex selection pressures. Hence, it may be inadvisable to explain melanism in terms of only one hypothesis (Wilson et al., 2001). Different selection pressures may result in patterns that are not consistent with the TMH.

Several previous studies have shown that the TMH can fail to explain relationships between melanism and environmental adaptations. In some cases, melanic morphs could be induced at relatively high temperatures or under a high level of incident radiation and were advantageous under warm conditions (Välimäki et al., 2015). The melanism of those species may arise from an interaction between thermal selection and other selective factors, especially over a wide geographical scale (Brakefield, 1985).

According to previous studies, the immunocompetence of darker individuals is usually greater than that of lighter ones (Armitage and Siva-Jothy, 2005). For example, the melanic morphs of *Spodoptera exempta* showed a significantly higher resistance to baculovirus than non-melanic morphs (Reeson et al., 1998), and melanic *Tenebrio molitor* showed lower mortality when exposed to a generalist entomopathogenic fungus (Barnes and Siva-Jothy, 2000). Additionally, melanic morphs of *Ephestia kuhuiella* were better able to inhibit the oviposition and larval development of parasitic wasps (Verhoog et al., 1996). Therefore, as we discussed above, we infer that the fitness advantages of melanic morphs at high temperatures may be explained by increased investment in melanin-based immunocompetence in the face of the greater risk of infection in a hotter environment (Roulin, 2014).

Although melanic morphs have multiple advantages, disadvantages still exist when the selection pressure changes. Melanin-based coloration is costly in terms of energy and other resources because it requires investment in production and maintenance (Roulin, 2015). A trade-off is needed between melanin-based coloration and other biological processes (Sheldon and Verhulst, 1996). This allocation problem may be solved by a distinctive pattern of investment in diverse aspects of body conditions in different color morphs that may result in different physiology or behavior. Thus, one morph is adapted to a given environmental condition but may exhibit maladaptation if this given environment is altered (Roulin, 2015). Hence, in the absence of certain selective factors, melanic morphs will have some disadvantages. For instance, darker morphs of pygmy grasshoppers (Orthoptera: Tetrigidae) usually select cool habitats due to the risk of overheating in hot environmental conditions (Ahnesjö and Forsman, 2006). Another example is the hypothesis that dark-colored damselfly larvae will normally be cryptic in their environment but will have a high risk of being preyed upon when observed against a white background (Johansson and Nilsson-Örtman, 2013).

The studies cited above identified potential selective disadvantages affecting melanic morphs under certain conditions. For this reason, melanism can at times be a cue for mate choice because it signals environment-dependent fitness to potential mates. For example, females of *Harmonia axyridis* prefer succinea-form males to melanic males in the spring generation due to the disadvantages experienced by melanic morphs during the summer (Su et al., 2013). Similarly, we infer that female *S. procerus* will also have their own mating preferences based on fitness variation in different environments although the melanic pattern found in the species is opposite to that expected from the THM. We consider that this preference may influence the rate of successful mating in *S. procerus* under different temperatures and photoperiods.

In summary, we found that photoperiod, in addition to temperature, is an environmental factor that influences melanism in *S. procerus*. Additionally, the adult longevity, fecundity, mating rate, and hatching rate of *S. procerus* were examined in the present study. Our results suggest that melanic morphs enjoy advantages in hot environments and under long photoperiods, whereas non-melanic morphs can adapt more successfully to low temperatures and short photoperiods. These results did not support the TMH. In view of the demonstrated differences in fitness between the two studied color morphs, we infer that the frequency of melanic morphs will increase as a result of continued increase in temperature associated with future global warming. The melanism of *S. procerus* appears in the adult stage but is a response to the environmental pressure experienced by the nymph. However, the mechanisms by which stressors in the nymphal or larval stage affect the adult stage remain unclear (Debecker et al., 2015). Additionally, it may be worthwhile to explore the genetic regulation of melanism to clarify the effect of heredity on the body color of *S. procerus*.

## Ethics statement

Our work conforms to the legal requirements of the country in which it was carried out.

## Author contributions

HY and JL: conception and design of research; HY and QS: performed experiments; HY: analyzed data; HY, QS, MS, JK, and JL: interpreted results of experiments; HY, MS: prepared figures; HY, MS: drafted manuscript; HY, MS, and JL: edited and revised manuscript; HY, QS, MS, JK, and JL: approved final version of manuscript.

### Conflict of interest statement

The authors declare that the research was conducted in the absence of any commercial or financial relationships that could be construed as a potential conflict of interest.
